# Diet Segregation between Cohabiting Builder and Inquiline Termite Species

**DOI:** 10.1371/journal.pone.0066535

**Published:** 2013-06-21

**Authors:** Daniela Faria Florencio, Alessandra Marins, Cassiano Sousa Rosa, Paulo Fellipe Cristaldo, Ana Paula Albano Araújo, Ivo Ribeiro Silva, Og DeSouza

**Affiliations:** 1 Departamento de Entomologia, Lab Termitologia, Universidade Federal de Viçosa, Viçosa, Minas Gerais, Brazil; 2 Departamento de Bioquímica, Universidade Federal de Santa Catarina, Curitibanos, Santa Catarina, Brazil; 3 Department of Biology, Pennsylvania State University, University Park, Pennsylvania, United States of America; 4 Faculdade de Engenharia, Universidade do Estado de Minas Gerais, João Monlevade, Minas Gerais, Brazil; 5 Infochemicals Research Team, Institute of Organic Chemistry and Biochemistry, Prague, Czech Republic; 6 Núcleo de Ecologia, Universidade Federal de Sergipe, São Cristóvão, Sergipe, Brazil; 7 Departamento de Solos, Laboratório de Isótopos Estáveis, Universidade Federal de Viçosa, Viçosa, Minas Gerais, Brazil; Fundação Oswaldo Cruz, Brazil

## Abstract

How do termite inquilines manage to cohabit termitaria along with the termite builder species? With this in mind, we analysed one of the several strategies that inquilines could use to circumvent conflicts with their hosts, namely, the use of distinct diets. We inspected overlapping patterns for the diets of several cohabiting Neotropical termite species, as inferred from carbon and nitrogen isotopic signatures for termite individuals. Cohabitant communities from distinct termitaria presented overlapping diet spaces, indicating that they exploited similar diets at the regional scale. When such communities were split into their components, full diet segregation could be observed between builders and inquilines, at regional (environment-wide) and local (termitarium) scales. Additionally, diet segregation among inquilines themselves was also observed in the vast majority of inspected termitaria. Inquiline species distribution among termitaria was not random. Environmental-wide diet similarity, coupled with local diet segregation and deterministic inquiline distribution, could denounce interactions for feeding resources. However, inquilines and builders not sharing the same termitarium, and thus not subject to potential conflicts, still exhibited distinct diets. Moreover, the areas of the builder’s diet space and that of its inquilines did not correlate negatively. Accordingly, the diet areas of builders which hosted inquilines were in average as large as the areas of builders hosting no inquilines. Such results indicate the possibility that dietary partitioning by these cohabiting termites was not majorly driven by current interactive constraints. Rather, it seems to be a result of traits previously fixed in the evolutionary past of cohabitants.

## Introduction

An efficient strategy for organisms which depend on nesting is to inhabit the nest of another species, because this avoids building costs while keeping the benefits of such structures. It is not surprising, therefore, that many inquiline species are spread throughout virtually all groups of animals. An intriguing issue is how invaders deal with potential conflicts with the builder, especially if invaders and builders cohabit, as frequently occurring with termite inquilines and their termite hosts. Here we provide evidence that inquilinism in certain termite nests seems to be eased by the use of conflict-avoiding strategies on the part of inquilines.

Examples of nest invaders include, but are not restricted to, nest-usurping woodpeckers, cuckoos and cowbirds [1], [2], joint nesting salamanders [3], inquiline bumblebees [4], and social parasitic butterflies [5]. In termite nests, intruders range from vertebrates such as birds [6] and bats [7] to a wide variety of arthropods [8], [9]. Most commonly, these assemblages are composed of a termite species that builds and maintains the nest, plus entire invertebrate food webs [10], [11] whose members are generally referred to as termitophiles. A particular subset of these is formed by termites that inhabit termite nests and may contribute to either nest maintenance or nest decay [12], the so called inquilines [13]–[17].

Inquiline termites form a particular group of invaders because, as their hosts, inquilines are detritivores. Risks imposed by inquiline termites are therefore rather distinct from those imposed, for instance, by predatory cohabitants such as larvae of elaterid beetles in termitaria [18], or the larvae of *Microdon* flies [19] and Lycaenidae butterflies [5] in ant nests. The absence of predation risks by no means implies the absence of trouble to the builder: negative interactions are still bound to arise if inquilines, e.g., feed on stored products or on the lining of the nest walls to a degree that requires constant replenishment or repair by the builders. At the very least, contests could be triggered when inquilines use a space originally built for the builder’s nestmates.

Dealing with such conflicts so that they represent bearable costs to the builder is key to the stability of cohabitation over ecological and evolutionary time. Therefore, a plausible hypothesis is that inquiline selection favours the adoption of strategies to minimise costs to the builder, which can be achieved by inflicting low loss or offsetting losses with an associated benefit. A wide range of strategies fulfil such aims, among which segregation of feeding resources is an obvious example of conflict avoidance. A possibility that cannot be excluded is that inquilinism is based on non-interactive processes: opportunistic inquilines occupy abandoned parts of termitaria and remain there unnoticed by the builders. In this case, the relationship could be evolutionarily stable because the use of such spaces would not be deleterious to the builder but would enhance the inquilines’ fitness through reduction of their own building costs.

In the present study we analysed the coexistence of termite builders and inquiline species in the same termitarium, in the field, with a focus on one of the mechanisms that could explain this interaction: the diet use by the species involved. To this end we evaluated diet coincidence between two builder termite species and 12 associated inquiline species, inspecting the stable isotopic signature of individuals from 14 termite nests in a savannoid ecosystem (*cerrado*) in South-eastern Brazil. Our rationale was that the diet of inquilines should differ from that of builders and the difference can be inferred from distinct 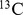/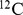 and 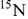/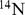 ratios for the termites. As a null hypothesis we consider that if invasion of these termitaria occurred merely by chance, without any evidence of past or present interaction, we would not find any consistent diet pattern for builder and inquiline species. In short, we argue here that one of the reasons for the coexistence of these builders and inquilines is that diet segregation minimises negative interactions and favours cohabitation in the same termitarium.

## Materials and Methods

### Ethics Statment

All necessary permits were obtained for the described study, which complied with all relevant regulations of Brazil. This includes collecting and transportation permits from IBAMA (The Brazilian Institute for the Environment and Renewable Natural Resources), permission from EMBRAPA (The Brazilian Enterprise for Agricultural Research) to conduct the study on their site, as well as tacit approval from the Brazilian Federal Government implied by granting the authors the post of Scientific Researcher.

### Definition of Terms

The term “termitarium” is used here to denote the physical epigeic structure built by termites (for taxonomic status see [20], [21]). We use “mound” and “nest” as synonyms of termitarium. “Colony” denotes the assemblage of individuals of a given species living and cooperating within the nest. “Coexistence” and “cohabitation” are used as synonyms and refer to the simultaneous occurrence of colonies of different termite species within a given termitarium, without implication of reciprocal positive or negative influences.

Diets exploited by termites were inferred from concentrations of stable carbon and nitrogen isotopes in termite bodies obtained by measuring 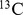/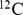 and 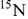/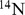 ratios. Termites from the same colony may forage on distinct lignocellulose sources with distinct degrees of decomposition. Therefore, the diet of a termite colony is characterized here by a set of 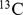/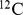 and 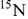/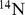 pairs obtained from several individuals from the same colony, this set circumscribing a bidimensional space in a Cartesian plot whose axes represent the concentration of such isotopes in termite bodies.

### Study Site

The study was carried out in the Brazilian *cerrado*, an environment physiognomically but not floristically similar to a savannah, near the town of Sete Lagoas (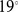
27′ S, 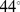
14′ W, altitude 800–900 m above sea level), Minas Gerais State, South-eastern Brazil. In Köppen’s classification, the study area has an Aw climate (equatorial with dry winter) [22]. The total precipitation in 2008 was 1607 mm and the mean monthly temperature ranged from 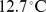 to 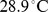 [23]. Fire often occurs naturally in the *cerrado* and the termites [24] and other organisms [25] that live there tolerate fire or depend on it to survive. Epigeous termitaria are a common feature of such an environment and inquilines frequently inhabit these termite mounds [26].

### Sampling

We sampled, from 24 to 28 July 2008 (7∶30–16∶00 h), 14 termitaria whose builder colonies were still alive and (apparently) healthy. These termitaria showed no sign of damage, were epigeic, and were easily removed from the soil without breaking its hypogeic portion. The termite builder species studied, *Velocitermes heteropterus* and *Constrictotermes cyphergaster* (both Termitidae: Nasutitermitinae), do not normally build termitaria presenting a significant hypogeic portion. It is worth noting that *C. cyphergaster*, which typically builds arboreal nests, can also build epigeous ones [27]. The termitaria were removed from the field, put into plastic bags, labelled, and taken to the laboratory. The vegetation and landscape were similar around all the termitaria sampled.

Once in the lab, the entire termitaria were carefully inspected to extract individuals using soft entomological forceps. Individuals from the same species grouped together were considered as belonging to the same cohabitant colony. Duplicate samples were taken from these cohabitant colonies, one for taxonomic identification and the other for isotopic analyses.

Specimens used for identification were preserved in 80% alcohol, labelled, and subsequently identified to species (or morpho-species) level according to Mathews [12] and literature referred to by Constantino [28]. Identifications were confirmed by comparison with the termite collection of the Entomological Museum of the Federal University of Viçosa (MEUV), where voucher specimens were deposited.

The builder species of each termitarium was determined by matching the termitarium physical traits with previous published accounts [12], [29] regarding size, geometric form, composition (soil or carton), wall texture, and wall hardness. In addition, builders tend to be far more abundant inside their termitarium than any inquiline.

Inquilines were identified as species whose colonies presented individuals of distinct instars, indicating that reproductive pairs were active and the colony was integrated in the environment. Some inquiline colonies were not populous enough to supply a minimum biomass of workers for isotopic analyses so their diet patterns were not mapped (these are denoted by ‘o’ for others in Table 1).

**Table 1:** Termite (morpho)species cohabiting termitaria in a ‘cerrado’ ecosystem.

	Termitaria													
Species or morpho-species	v1	v2	v3	v4	v5	v6	v7	c1	c2	c3	c4	c5	c6	c7
**RHINOTERMITIDAE**														
**Heterotermitinae**														
*Heterotermes longiceps*		o						i						
*Heterotermes tenuis*					o									
**TERMITIDAE**														
**Apicotermitinae**														
*Anoplotermes* sp.1		i	i											
*Anoplotermes* sp.2		i												
*Anoplotermes* sp.3						i								
*Grigiotermes* sp.1			i											
*Grigiotermes* sp.2					i									
*Grigiotermes* sp.3		o												
**Nasutitermitinae**														
*Constrictotermes cyphergaster*								b	b	b	b	b	b	b
*Nasutitermes coxipoensis*					o									
*Nasutitermes* sp.1					o									
*Nasutitermes* sp.2							o							
*Subulitermes* sp.						o								
*Velocitermes heteropterus*	b	b	b	b	b	b	b							
**Syntermitinae**														
*Cyranotermes timuassu*							o							
*Labiotermes brevilabius*	i	i				i								
*Procornitermes araujoi*							i							
*Silvestritermes euamignathus*				i			i							
**Termitinae**														
*Inquilinitermes microcerus*									i	i	i			
*Neocapritermes* sp.	o					i								
*Orthognatotermes* sp.							o							
*Spinitermes trispinosus*			i				o							
Number of (morpho)species	3	6	4	2	5	5	7	2	2	2	2	1	1	1

‘b’ = termitarium's builder; ‘i’ = inquiline species whose high abundance allowed isotopic analyses; ‘o’ = other inquilines, whose low abundance prevented isotopic analyses. Each column is a single termitarium.

### Stable Isotope Analysis

We used stable isotope concentrations to infer diet because the isotopic composition of the body of an animal reflects the food consumed and assimilated during its lifetime [30], [31]. Within a given environment, comparatively higher 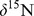 values indicate a termite diet biased towards more humified organic matter, whereas lower values point to a less decomposed, even xylophagous diet. Bourguignon *et. al.* [32] presented a practical example of such a classification.

Termite workers of each species in the termitaria were sorted, when possible, into 10 subsamples, each with a sufficient number of individuals to obtain a dry biomass of 1.5 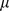
g for full-body isotopic analysis. Colonies meeting this criterion are denoted by ‘b’ (for builders) and ‘i’ (for inquilines) in Table 1. We used only workers for stable isotope analysis, not only because these are the most abundant individuals in a termite colony but also because they forage and feed other castes in the colony [33]. This procedure also eliminated any possible intercaste effects on isotopic values [34].

Each subsample was placed in a vial with distilled water and was immediately frozen until the analyses could be performed. Water was removed by freeze-drying for approximately 48 h to dehydrate the termites, prevent decomposition and maintain the original 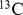/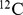 and 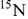/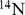 ratios. The subsamples were then ground with a mortar and pestle and sieved through a 100-mesh sieve. Carbon and nitrogen isotope ratios were measured for each subsample independently, using an isotope ratio mass spectrometer (IRMS, ANCA-GSL 20–20, SerCon, UK) in the Laboratory of Stable Isotopes, Soils Department, Federal University of Viçosa (UFV). The analytical precision was estimated to be 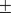
0.1‰ for carbon and 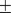
0.2‰ for nitrogen. The natural abundance of 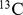 and nitrogen 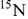 is expressed as per thousand (‰) deviation from an international standard(belemnite of the Pee Dee Formation in South Carolina, USA (PDB) for carbon and atmospheric nitrogen (air) for nitrogen). The ratios of the heavy (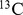 or 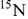) to the light isotope (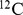 or 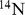), typically corresponding to rare and abundant isotopes are hereafter referred to as “isotopic ratios” [35] and are referenced by 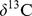 and 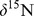.

### Data Analysis

Diet limits were statistically defined as Bayesian standard ellipses plotted around pairs of 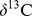 and 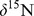 points representative of termites’ diet space, such ellipses being to bivariate data as standard deviation is to univariate data. Because these ellipses define the statistical limits for the dimensions of each diet, overlapping ellipses indicate statistically indistinguishable diet spaces. Ellipses and associated metrics were calculated using siber routines [36] from siar package [37], under R statistical computing environment [38].

Ellipses were estimated according to three distinct and complementary views of the dataset. Initially, a single ellipsis was estimated for the whole community of cohabitants within a given termitarium, thereby allowing comparisons among whole termitaria across the sampled region. Overlapping ellipses would indicate similarity between diets among termitaria in spite of their spatial distribution over the sampled region. Then, the data relative to the full set of inquilines of a given builder species were amassed (across all termitaria) in a single ellipsis, thereby allowing comparisons with the single ellipsis of the respective builder species, also amassed across all termitaria. This allowed to infer general patterns for diet spaces of inquilines *versus* builders. Finally, individual ellipses were plotted for each cohabitant within each termitarium, thereby allowing diet comparisons between builders and their respective inquilines within a given termitarium.

To infer on interactive processes regulating diet segregation we checked for correlation between the dimensions of diet spaces of cohabitants within each termitarium. If inquilines dynamically expand their diets at the expense of their host’s diets (or vice-versa), the dimension of their respective diet spaces across all termitaria should correlate negatively. Accordingly, diet spaces of builders living alone should be larger than those of builders cohabiting with inquilines. Analyses were carried out using Generalized Linear Modelling under normal errors followed by residual analyses to confirm the model suitability and the choice of error distribution. Initially, a subset of the data containing only termitaria having both, builders and inquilines, was subjected to a model in which the area of the builder's ellipsis (y-var) was correlated with the respective area of the ellipsis formed by their respective inquilines taken together (x-var). The identity of the builder entered the model as a covariate, both as a single term and as part of the first order interaction. Another independent model compared the average area of the builder's ellipsis (y-var) between termitaria with and without inquilines (x-var). This was only possible on termitaria built by *C. cyphergaster* because for those both instances of the x-variable were available. Models were simplified by deleting non-significant terms (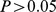) from the initial model according to their complexity, starting with the most complex term, following recommendations by Crawley [39].

## Results

### Species Distribution among Termitaria

A survey carried out in the study area revealed that termitaria were 4.4 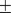 1.7 m (mean 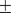 SD) apart from their four nearest neighbouring termitaria. This survey included, but was not restricted to, the termitaria studied here.

Some 20 species of termite inquilines were found in the termitaria, of which 12 species presented individuals enough to be analysed isotopically. A total of 13 species occurred only once (Table 1) and seven occurred in two or more termitaria. Termitaria sheltered between zero and six inquiline species. Termitaria of *V. heteropterus* sheltered between one and six inquiline species at once, whereas termitaria of *C. cyphergaster* housed between zero and one inquiline species.

*Heterotermes longiceps* was the sole inquiline species found in termitaria of both builder species, but it was neither frequent nor abundant: only two very small colonies were recorded, the largest of which comprised approximately 40 individuals. The remaining 19 inquiline species were not shared between builder species, suggesting species-specific differences in the ability to coexist with other species. Supporting such a trend, *Inquilinitermes microcerus* did not occur in termitaria of *V. heteropterus* but it was found only in termitaria of *C. cyphergaster*. This is in line with previous reports that *I. microcerus* is an obligatory inquiline of *C. cyphergaster* [12].

The 14 termitaria studied housed 29 inquiline colonies along with the builder colony (‘i’+‘o’ in Table 1), of which 18 colonies presented individuals enough to be analysed isotopically (‘i’ in Table 1). Termitaria housing multiple colonies showed no evidence of more than a single colony of a given cohabitant species.

### Diet Segregation

**Figure 1:**
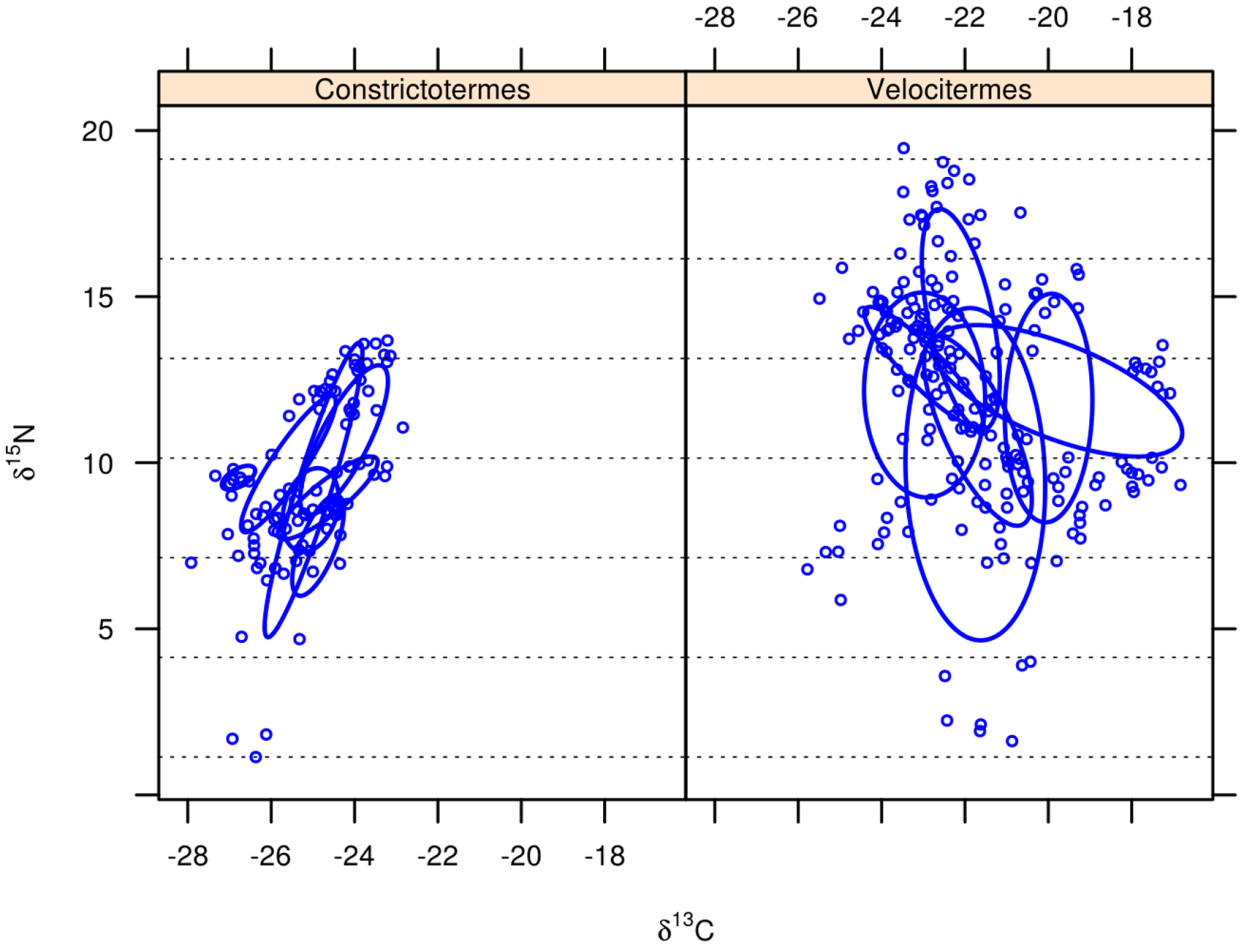
Overall summary of diet spaces of termite communities cohabiting termitaria, as inferred from their isotopic profile. Each panel depicts a set of termitaria of a given builder (*Constrictotermes cyphergaster* or *Velocitermes heteropterus*). Axes represent the concentration of stable carbon and nitrogen isotopes in termite bodies. Each dot refers to a group of termite workers weighing 1.5 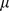
g. Each Bayesian standard ellipsis represents a single termitarium. Dotted lines parallel to x-axis define 3‰ 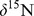 intervals thought to correspond to trophically distinct positions. Both, inquiline and builder species are included. See Table 1 for termite species identities.

**Figure 2:**
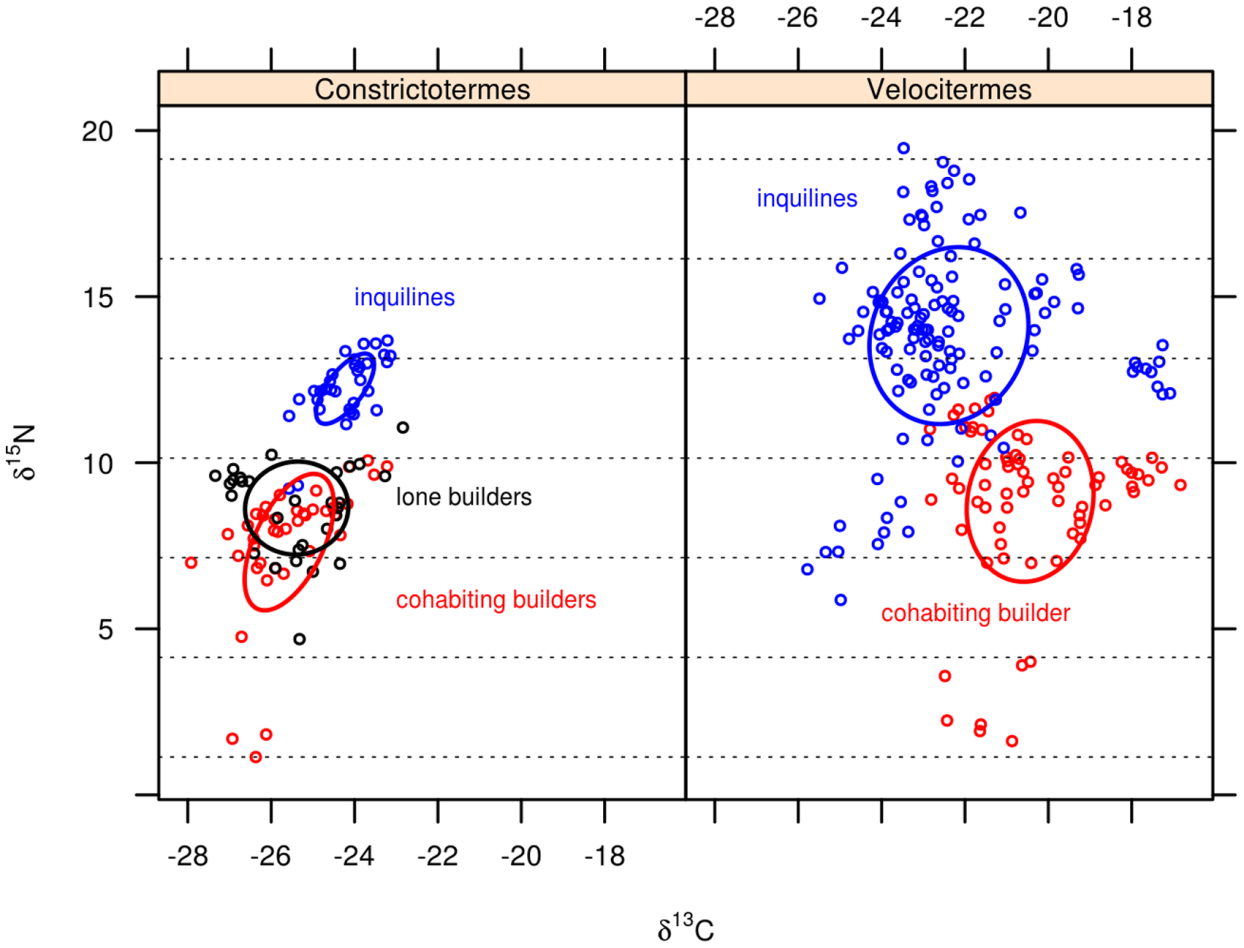
Overall summary of diet spaces of builder and inquilines termites, as inferred from their isotopic profile. Each panel depicts a set of termitaria of a given builder (*Constrictotermes cyphergaster* or *Velocitermes heteropterus*). Axes represent the concentration of stable carbon and nitrogen isotopes in termite bodies. Each dot refers to a group of termite workers weighing 1.5 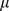
g. Each Bayesian standard ellipsis represents the full set of builders or inquilines amassed across all termitaria studied. Dotted lines parallel to x-axis define 3‰ 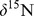 intervals thought to correspond to trophically distinct positions. See Table 1 for termite species identities.

**Figure 3:**
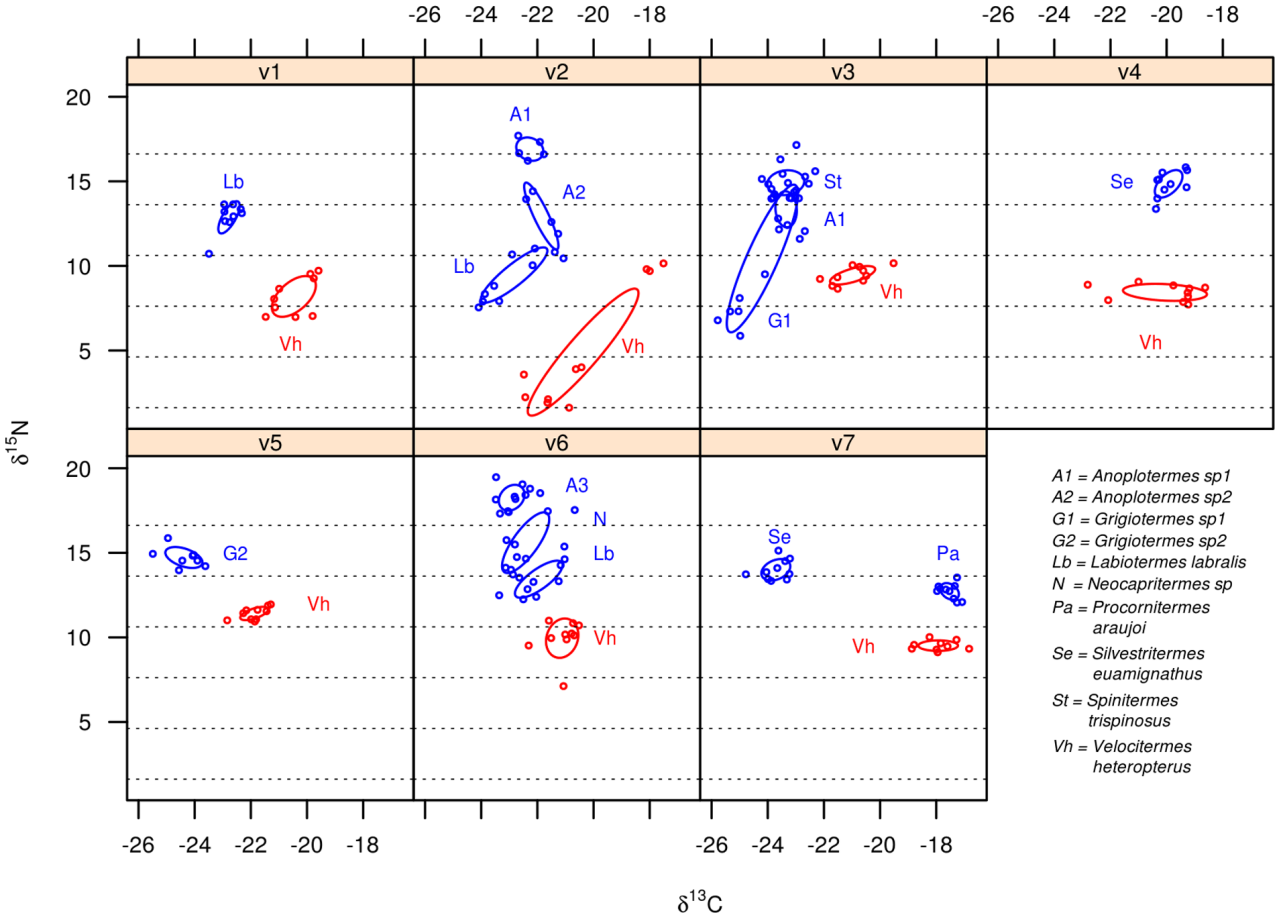
Diet spaces of termite species coexisting in termitaria built by *Velocitermes heteropterus*, as inferred from their isotopic profile. Each panel represents a single termitarium. Axes represent the concentration of stable carbon and nitrogen isotopes in termite bodies. Each dot refers to a group of termite workers weighing 1.5 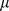
g. Bayesian standard ellipses refer to the builder and its inquiline termite species. Dotted lines parallel to x-axis define 3‰ 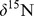 intervals thought to correspond to trophically distinct positions.

**Figure 4:**
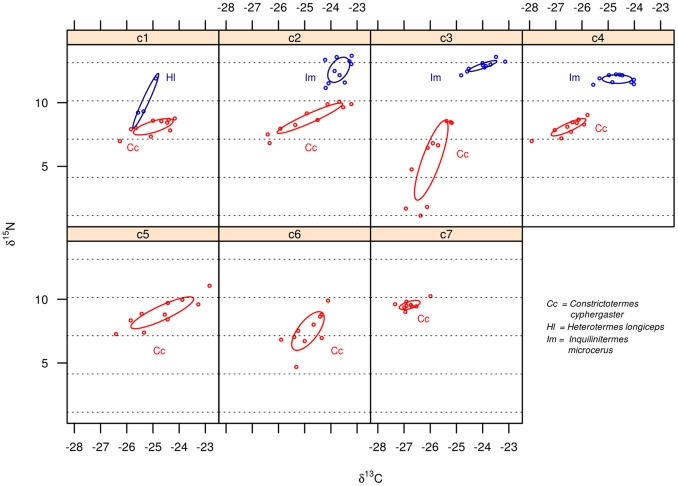
Diet spaces of termite species coexisting in termitaria built by *Constrictotermes cyphergaster*, as inferred from their isotopic profile. Each panel represents a single termitarium. Axes represent the concentration of stable carbon and nitrogen isotopes in termite bodies. Each dot refers to a group of termite workers weighing 1.5 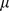
g. Bayesian standard ellipses refer to the builder and its inquiline termite species. Dotted lines parallel to x-axis define 3‰ 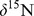 intervals thought to correspond to trophically distinct positions.

Termitaria overlapped each other regarding the overall diet space of their community of cohabitants (Fig. 1) indicating that, in average, communities exploited rather similar diets despite being confined to distinct termitaria. A single *C. cyphergaster* nest did not overlap the others (Fig. 1, leftmost ellipsis, corresponding to nest c7). This nest is devoid of inquilines and its detachment was not strong enough to scramble the statistical non-overlaping trend presented by the other nests, as it is shown in Fig. 2, “builder alone”. The diets of builder and inquiline species never overlapped (Fig. 2, 3 and 4). This diet segregation tended to be majorly driven by 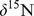 isotopic dimension, with inquilines occupying a higher trophic position than builders (Fig. 2), albeit still within the detritivore level (horizontal dotted lines in all figures denote changes in trophic position, as it is generally agreed that trophically distinct organisms would differ by 3‰ in 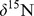 [40]).

There was also a general trend for diet segregation among inquiline species within termitaria, with only a single case of overlap out of 14 nests (Fig. 3, v3). Diet segregation among inquilines was also most obvious in the 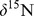 dimension.

### Diet Shrinkage

There was no correlation between the areas of the builder’s diet space and that of its inquilines (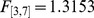, 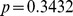). Accordingly, the diet areas of *C. cyphergaster* builders hosting inquilines were in average as large as the areas of builders hosting no inquilines (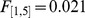, 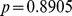). This seems to lend support to the notion that inquilines and builders do not interfere to each other in terms of their diet.

## Discussion

Differentiation in resource use has been considered one of the main mechanisms that facilitates species coexistence (for a comprehensive historical account, see [41]) that includes communities of plants [42], [43], fish [44], and insects [45], [46]. In communities of termites, interactions with respect to food resources have been identified as an important regulating factor; examples include species assemblages from the African savannah [47], [48] and the South American tropical rainforest [32], [34].

While dietary shifts seem to affect the coexistence of termite species in environments delimited by permeable borders, patterns of interaction with respect to diet are virtually unknown for termite species assemblages circumscribed by discrete physical barriers (but see [49] for competing insular termite populations), especially those cohabiting the same termitarium.

Such spatially confined populations represent suitable scenarios for studying dietary shifts as determinants of species coexistence. Because barriers restrict spatial adjustments that could preclude species interactions, the importance of dietary adjustments may in turn be amplified. In fact, for the termite builder–inquiline assemblages studied here, feeding resource segregation appears to be typical, if not the determinant, of cohabitation in the same termitarium. Diet spaces for inquilines never overlapped host’s spaces at both, regional (i.e. the sampled environment) and local (termitarium) scales (Figs. 2, 3 and 4). Mechanisms behind this diet segregation could include (i) local differences in the suitability and availability of resources, including predation constraints [50], [51], so that each set of cohabitants in a termitarium has access to a particular diet; and (ii) local-scale interspecific tradeoffs [52] leading to diet partitioning along a trophic continuum within the termitarium.

The fact that inquiline-bearing termitaria presented strong overlap regarding the overall diets of their cohabiting communities (Fig. 1) seems to point out that cohabitants had access to similar resources, and that is reinforced by the close proximity of all nests (in average 4.4 m apart). Additionally, consistent patterns of non-overlapping diets between builders and inquilines and among inquilines across all termitaria (Figs. 2, 3 and 4) seem to weaken the hypothesis of local differences in resources in favour of the trade-off hypothesis, even though these are not necessarily mutually exclusive.

Segregation in resource use on its own does not imply species interactivity since species can be assembled by chance events [53]. However, as well as the consistent differences observed for their actual diets (Fig. 3 and 4), the cohabiting termites studied here did not seem to be assembled at random (Table 1). Rather, inquiline species of *V. heteropterus* did not seem to be able to live in termitaria of *C. cyphergaster* and vice versa. This is reinforced by the presence of the obligatory inquiline *I. microcerus* [12], [29], [54], which was only found in *C. cyphergaster* nests. In fact, ocupation of *C. cyphergaster* nests by *I. microcerus* is not believed to occur at random but to depend on host/nest features [16]. Thus, inquiline occupation in these termitaria is likely to be related to the intrinsic characteristics of the species involved rather than being a simple chance event.

Diet segregation under such a deterministic scenario could result from feeding resource competition but the absence of overlap between the overall diet spaces of inquiline and builders would challenge this idea, because even when not sharing the same termitarium and hence not subject to potential conflicts, the diet of inquiline species never overlapped that of host species (Fig. 2). Indeed, the mere fact that inquilines did exploit *distinct* diets makes it risky to advocate some link between the observed segregation and contemporary competitive interactions. It seems therefore that dietary partitioning by cohabitants was not majorly driven by interactive constraints, a hypothesis also supported by the absence of correlation between the diet space areas of builder and inquiline species within termitaria (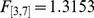; 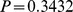), where interactions would be highly likely. This is further supported by the fact that the average diet areas of *C. cyphergaster* builders did not expand significantly in the absence of inquilines (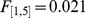, 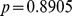). In other words, interspecific tradeoffs as a force driving termite inquilinism in this system would more likely to have occurred – if at all – in the evolutionary past rather than in the contemporary ecological time frame (the ‘ghost of competition past’ [55]). An alternative and perhaps more conservative view is that current inquiline species are descended from specialist lineages, and never conflicted with the dietary requirements of their hosts.

Despite being not able to distinguish between the hypotheses of past interspecific trade-off *versus* pre-adaptations favouring specialization, our data reinforce both hypotheses over a hypothesis focusing on current competition. Although still within the detritivore trophic level, inquiline and builder species never shared the same trophic position (Figs 3 and 4) and were sometimes as much as four full positions apart (taking each trophic step as 3‰ units in 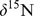, as in de Visser *et al.* [10]). Since inquiline species are obviously not predators but detritivores, such disparate trophic positions may indicate that they in fact feed on materials far more decayed than those used by the builder species. These could include stored organic material, the hosts faeces and dead bodies, and the lining of the termitarium walls, which is also composed of faeces. Although still open to investigation, this assumption is in line – at least regarding *I. microcerus*– with previous reports by Noirot [56] and Mathews [12] and recent evidence by Bourguignon *et al.* [32].

Diet differences were also observed among most inquiline species cohabiting the same termitarium; those that actually differed being arranged in stepwise trophic positions. It is possible that a trophic chain was established, with one inquiline species feeding on the by-products of its host, another feeding on the excreta and remains of this inquiline, and so on. Alternatively, inquiline species could selectively feed on distinct parts of the nest, and thus would have distinct 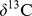 and 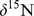 inputs. Termites are indeed able to feed selectively in the field [51] and can select soil particles from distinct layers to build specific mound structures, which in turn exhibit distinct C and N contents [57] most likely with characteristic 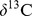 and 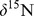 values. This would explain not only the consistent differences observed between builder and inquiline species regarding 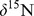 dimensions of their diets, but also the fact that diets of inquilines, albeit still distinct, differed sometimes in a single dimension and sometimes in both. In other words, under this scenario, inquiline species and their host would differ less markedly in 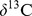 than in 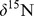 (Figs. 1, 3 and 4) because by feeding on specific parts of the nest, inquiline species have access to a subset of the carbon resources collated by their host along with selected soil particles that are trophically distinct because they are cemented with the host’s faeces. The possibility that a given inquiline species could also differ from the builder and other inquiline species by foraging for distinct food outside the nest [12] remains to be considered. All in all, this would only reinforce the diet segregation patterns observed here.

In summary, we found evidence that, at least for the system at hand, cohabitation of termite species in the same termitarium was related to diet segregation that did not seem to be majorly constrained by interspecific interactions for food. Rather, inquilines exploited diets not used by their host, thereby circumventing conflicts over use of feeding resources.
